# A hybrid self-attention deep learning framework for multivariate sleep stage classification

**DOI:** 10.1186/s12859-019-3075-z

**Published:** 2019-12-02

**Authors:** Ye Yuan, Kebin Jia, Fenglong Ma, Guangxu Xun, Yaqing Wang, Lu Su, Aidong Zhang

**Affiliations:** 1 College of Information and Communication Engineering, Beijing University of Technology, Beijing, China; 2 Beijing Laboratory of Advanced Information Networks, Beijing, China; 3 Beijing Key Laboratory of Computational Intelligence and Intelligent System, Beijing University of Technology, Beijing, China; 4 Department of Computer Science and Engineering, State University of New York at Buffalo, Buffalo, NY USA; 5 Department of Computer Science, University of Virginia, Charlottesville, NV USA

**Keywords:** Attention mechanism, Deep learning, Sleep stage classification, Polysomnography, Multivariate time series

## Abstract

**Background:**

Sleep is a complex and dynamic biological process characterized by different sleep patterns. Comprehensive sleep monitoring and analysis using multivariate polysomnography (PSG) records has achieved significant efforts to prevent sleep-related disorders. To alleviate the time consumption caused by manual visual inspection of PSG, automatic multivariate sleep stage classification has become an important research topic in medical and bioinformatics.

**Results:**

We present a unified hybrid self-attention deep learning framework, namely HybridAtt, to automatically classify sleep stages by capturing channel and temporal correlations from multivariate PSG records. We construct a new multi-view convolutional representation module to learn channel-specific and global view features from the heterogeneous PSG inputs. The hybrid attention mechanism is designed to further fuse the multi-view features by inferring their dependencies without any additional supervision. The learned attentional representation is subsequently fed through a softmax layer to train an end-to-end deep learning model.

**Conclusions:**

We empirically evaluate our proposed HybridAtt model on a benchmark PSG dataset in two feature domains, referred to as the time and frequency domains. Experimental results show that HybridAtt consistently outperforms ten baseline methods in both feature spaces, demonstrating the effectiveness of HybridAtt in the task of sleep stage classification.

## Background

Sleep is a complicated biological process and plays an essential role in health. Sleep occurs in cycle and involves different sleep stages, helping restore functions of body and mind, such as immune, nervous, skeletal, and muscular systems [1]. Unhealthy lifestyles and work-related stress may lead to sleep disturbances, which has become one of the serious issues in modern societies. Sleep disorders not only cause a reduction in physical performance during the day, but have negative effects on cognitive functions [2]. Moreover, some psychological and neurological diseases can also deteriorate normal sleep patterns [3]. Towards this end, in order to provide prevention and treatment of the sleep-related disorders, sleep stage analysis has garnered great interest among researchers in medical and bioinformatics recently.

In practice, physicians often use polysomnography (PSG) records to comprehensively analyze sleep [4]. PSG data contain multivariate physiological signals, such as electroencephalogram (EEG), electromyogram (EMG), electrocardiogram (ECG), and electrooculogram (EOG), in order to monitor different body regions. In particular, through visual inspection, each 30-s time slot of PSG data can be classified into different sleep stages by different rules. According to the standard Rechtschaffen and Kales (R&K) rules [5], for example, the sleep phase can be classified into stages as wakefulness, non-rapid eye movement (NREM) sleep, and rapid eye movement sleep. Among them, the NREM sleep is further subdivided into four sleep stages referred to as S1, S2, S3, and S4. However, it is extremely time-consuming and laborious for physicians to visually inspect long-term PSG records. In addition, identifying and analyzing sleep patterns also requires highly-trained professionals. Therefore, it is necessary to develop an automatic system capable of classifying sleep stages to enhance efficiency of PSG sleep analysis.

In recent years, various automatic sleep stage classification systems have been presented utilizing overnight PSG records [2, 3]. Several researchers focus on extracting different handcrafted features from multivariate PSG data to train an aggregated classifier. On one hand, different kinds of discriminative features, such as time-domain features [6, 7], frequency-domain features [8, 9], and other nonlinear measurements [10, 11], have been adopted to analyze the PSG data in each time slot. On the other hand, some well-known classifiers in machine learning, including support vector machine (SVM) [12, 13] and neural networks (NN) [14, 15], are employed to help identify the sleep stages. These methods advance the development of automatic sleep stage classification systems, but typically requires a significant amount of domain knowledge and would not guarantee consistent good performance using multi-stage training procedures to make all the components work together. Furthermore, the recent advances in deep learning allow researchers to improve classification performance by directly learning feature representations from the multivariate biosignals [16]. By constructing multi-layer neural networks in different way, some classic deep learning structures, such as deep belief networks (DBN) [17, 18], convolutional neural networks (CNN) [19–21] and recurrent neural networks (RNN) [22, 23], have been well applied in the task of sleep stage classification with promising results.

However, existing deep learning models lack a mechanism to extract comprehensive correlations of the multivariate PSG records, presenting a challenge to accurately classify sleep stages. Specifically, the complex correlations among PSG channels are important to recognize sleep patterns. For instance, the abnormal wake-up (i.e., wakefulness stage) in central sleep apnea is caused by the nervous system irregularities which trigger the heart abnormalities and muscles movements [24]. These correlated physiological conditions can be reflected from EEG, ECG, and EMG, respectively, which are helpful for sleep stage classification. Secondly, PSG data involve dynamic correlations across different timestamps (or time slots), which help identify informative events during sleep, such as irregular sleep-wake rhythm and sudden involuntary movement [25], to improve classification performance.

To this end, we propose HybridAtt, a deep learning framework with hybrid self-attention mechanism to classify sleep stages from the multivariate PSG inputs. The proposed hybrid self-attention mechanism is able to capture the dual correlations of PSG channels and timestamps by inferring their dependencies without any additional supervision. Moreover, a multi-view convolutional representation module is constructed to help the proposed attention mechanism fuse PSG data. We conduct cross-subject experiments in comparison with ten baseline methods, and demonstrate the effectiveness of our proposed HybridAtt model on a benchmark PSG dataset in two feature domains, referred to as the time and frequency domains. We summarize our main contributions as follows:

- We propose HybridAtt, an end-to-end hybrid self-attention deep learning framework for sleep stage classification using multivariate PSG records.
- HybridAtt explicitly extracts the dual correlations of PSG channels and timestamps by inferring their dependencies based on multi-view convolutional representations.
- We empirically show that HybridAtt consistently achieve best performance compared with ten baselines on a benchmark dataset under different feature domains.

## Methods

In this section, we introduce the technical details of our HybridAtt model with multivariate PSG inputs. We first describe the overall architecture and then detail the main components of HybridAtt.

### Model architecture

**Fig. 1:**
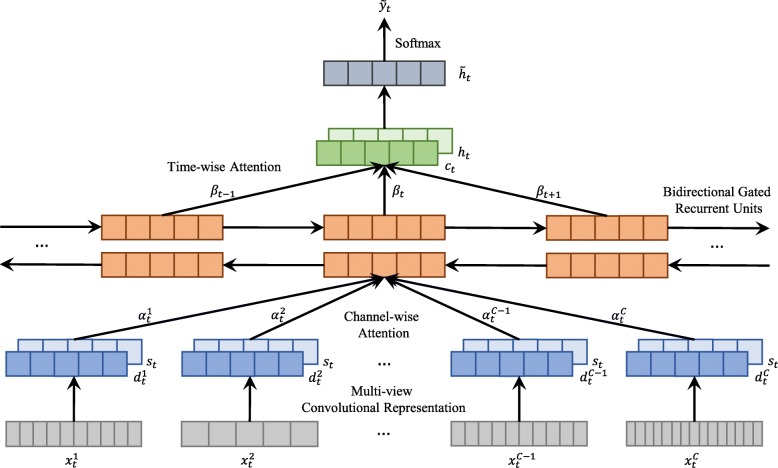
Main architecture of the HybridAtt model. The goal of HybridAtt is to capture dual correlations of PSG channels and timestamps by calculating the dependencies of their multi-view convolutional representations, in order to improve the performance of sleep stage classification using multivariate PSG records

Figure 1 presents the architecture of our proposed HybridAtt model. The goal of HybridAtt is to capture dual correlations of PSG channels and timestamps by calculating the dependencies of their multi-view convolutional representations, in order to improve the performance of sleep stage classification using multivariate PSG records. Formally, we assume that there are *M* multivariate PSG records with *T*^(*M*)^ timestamps, denoted as $\left \{\boldsymbol {X}_{1}^{(m)}, \boldsymbol {X}_{2}^{(m)}, \cdots, \boldsymbol {X}_{T^{(m)}}^{(m)}\right \}_{m=1}^{M}$. Each record ***X***_*t*_ at timestamp *t* contains a set of *C*-channel heterogeneous waveform vectors $\left \{\boldsymbol {x}_{t}^{1}, \boldsymbol {x}_{t}^{2}, \cdots, \boldsymbol {x}_{t}^{C}\right \}$ where $\boldsymbol {x}_{t}^{c} \in \mathbb {R}^{n^{(c)}}$. To learn informative features from the heterogeneous inputs, in our model, we first feed the input ***X***_*t*_ into a multi-view convolutional representation module to extract the channel-view hidden features $\boldsymbol {d}_{t}^{1:C}$ and global-view hidden features ***s***_*t*_, respectively. We then develop a channel-wise attention module to capture the complex channel correlations at each timestamp based on the learned multi-view features. Subsequently, a time-wise attention module, combined with bidirectional gated recurrent units (BGRU), is utilized to distinguish the dynamic correlations. Here we use ***h***_*t*_ and ***c***_*t*_ to denote the learned hidden state and context vector at timestamp *t*. Finally, we can further obtain an attentional hidden representation $\boldsymbol {\tilde {h}}_{t}$ to predict the label $\boldsymbol {y}_{t} \in \{0,1\}^{|\mathcal {C}|}$ where $|\mathcal {C}|$ is the unique number of categories related to sleep stages. The proposed model can be trained in an end-to-end fashion.

### Multi-view convolutional representation

In practice, the collected PSG data often tend to be heterogeneous, referred to different sample rates, signal strengths, and rhythm patterns. Inspired by the rapid development of multi-view deep learning [26–29], we propose to modify the CNN structure to preserve the unique characteristics of each biomedical channel during feature representation. Given the input $\boldsymbol {x}_{t}^{c}$ in the *c*-th channel at timestamp *t*, we use a 1-D channel-CNN encoder (i.e., CNN_*c*_) to derive its channel-view representation $\boldsymbol {d}_{t}^{c} \in \mathbb {R}^{p}$, as follows:

1 $$\begin{array}{@{}rcl@{}}  \boldsymbol{d}_{t}^{c} = \text{CNN}_{c}\left(\boldsymbol{x}_{t}^{c};\boldsymbol{\theta_{c}}\right), \end{array} $$

where ***θ***_***c***_ denotes the learnable parameter set of CNN_*c*_. Similarly, we utilize a 2-D global-CNN encoder (i.e., CNN_*g*_) to obtain a global-view representation $\boldsymbol {s}_{t} \in \mathbb {R}^{p}$ based on all the channels, as follows:

2 $$\begin{array}{@{}rcl@{}}  \boldsymbol{s}_{t} = \text{CNN}_{g}\left(\boldsymbol{x}_{t}^{1:C};\boldsymbol{\theta_{g}}\right), \end{array} $$

where ***θ***_***g***_ denotes the learnable parameter set of CNN_*g*_. Here we align the input dimension of each channel using linear interpolation to obtain a matrix input for Eq. (2).

**Fig. 2:**
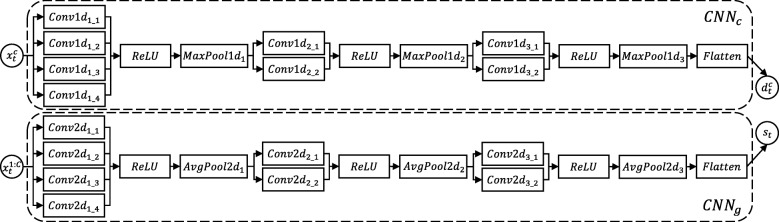
CNN Structure of the multi-view convolutional representation module in HybridAtt. Informative features can be well extracted from the heterogeneous PSG inputs, and hence can further help the hybrid attention mechanism capture dual correlations

In order to unleash the power of the multi-view convolutional representation module, we further polish the CNN structure design in our HybridAtt model, as shown in Fig. 2. The main design strategy consists of two aspects. First, the convolutional layer should cover multiple resolution scales since the waveform patterns of biosignals are related to different frequency modes [30]. Here we set different sizes of feature kernels in parallel to extract multi-scale features from biosignals. Second, CNN_*c*_ and CNN_*g*_ should focus on different characteristics of input data during feature learning. Towards this end, we guide these two encoders by setting max pooling for CNN_*c*_ to extract the most important features of different channels, and setting average pooling for CNN_*g*_ to retain more general information among all the channels. Taking the advantage of the multi-view structure, informative features with same dimensions can be well learned from the heterogeneous PSG inputs, and hence further help the hybrid attention mechanism capture the dual correlations.

### Hybrid self-attention mechanism

#### Channel-wise attention

In order to capture the complex correlations among PSG channels, we develop a channel-wise attention layer that is able to infer the importance of each channel based on the learned multi-view features, and fuse representations relied on more informative ones. Given the multi-view features $\boldsymbol {d}_{t}^{c}$ and ***s***_*t*_ obtained by Eqs. (1) and (2), we first compute a fusional rate $r_{t}^{c} \in \mathbb {R}$ for each channel *c* at timestamp *t*, inferring how much information carried by each CNN encoder should be fused. The formulation is as follows:

3 $$\begin{array}{@{}rcl@{}}  r_{t}^{c} = \sigma \left(\boldsymbol{W}_{rg}^{\top}\boldsymbol{s}_{t} + \boldsymbol{W}_{rc}^{\top}\boldsymbol{d}_{t}^{c} + b_{rc}\right), \end{array} $$

where $\boldsymbol {W}_{rg} \in \mathbb {R}^{p}, \boldsymbol {W}_{rc} \in \mathbb {R}^{p}$, and $b_{rc} \in \mathbb {R}$ are learnable parameters. Here we rescale $r_{t}^{c}$ into the range of [0,1] using sigmoid function *σ*(·) in Eq. (3). Then, we assign an attention energy $e_{t}^{c}$ for each channel *c* based on its fusional rate $r_{t}^{c}$, as follows:

4 $$\begin{array}{@{}rcl@{}}  e_{t}^{c} = \boldsymbol{W}_{ec}^{\top}\left(\left(1-r_{t}^{c}\right) \odot \boldsymbol{s}_{t} + r_{t}^{c} \odot \boldsymbol{d}_{t}^{c}\right)+b_{ec}, \end{array} $$

where $\boldsymbol {W}_{ec} \in \mathbb {R}^{p}$ and $b_{ec} \in \mathbb {R}$ are learnable parameters, and ⊙ denotes the element-wise multiplication operator. Given the attention energy, a channel-wise contribution score vector $\boldsymbol {\alpha }_{t} \in \mathbb {R}^{C}$ can be normalized using softmax function, as follows:

5 $$\begin{array}{@{}rcl@{}}  \boldsymbol{\alpha}_{t} = \text{Softmax}\left(\left[e_{t}^{1}, \cdots, e_{t}^{c},\cdots, e_{t}^{C}\right]\right). \end{array} $$

Each element $\alpha _{t}^{c}$ in the vector measures the importance of information carried by the *c*-th channel.

Accordingly, we use weighted aggregation to calculate the output vector of the channel-wise attention $\boldsymbol {\tilde {x}}_{t} \in \mathbb {R}^{2p}$ based on the contribution score vector ***α***_*t*_:

6 $$\begin{array}{@{}rcl@{}}  \boldsymbol{\tilde{x}}_{t}=\boldsymbol{s}_{t} \oplus \left(\sum\limits_{c=1}^{C}\alpha_{t}^{c} \odot \boldsymbol{d}_{t}^{c}\right), \end{array} $$

where ⊕ is the concatenation operator. In this way, our model can fully incorporate the multi-view information carried by both two feature views, and thus fuse more informative features from multivariate PSG records.

#### Time-wise attention

To capture the dynamic correlations across timestamps, the aforementioned attention strategy can be employed as well, namely time-wise attention. Given the learned vector sequence from $\boldsymbol {\tilde {x}}_{1}$ to $\boldsymbol {\tilde {x}}_{T}$, we derive the hidden state $\boldsymbol {h}_{t} \in \mathbb {R}^{2q}$ through a 2-layer BGRU [31], as follows:

7 $$\begin{array}{@{}rcl@{}} \boldsymbol{h}_{1:T}=\text{BGRU}(\boldsymbol{\tilde{x}}_{1:T};\boldsymbol{\theta}_{r}), \end{array} $$

where ***θ***_*r*_ is the learnable parameter set of BGRU. Here the hidden state ***h***_*t*_ at timestamp *t* is obtained by concatenating the forward hidden vector $\overrightarrow {\boldsymbol {h}}_{t} \in \mathbb {R}^{q}$ and the backward hidden vector $\overleftarrow {\boldsymbol {h}}_{t} \in \mathbb {R}^{q}$ in BGRU.

Subsequently, we can reformalize the attention strategy from Eqs. (3) to (5), to compute the time-wise contribution score vector $\boldsymbol {\beta }_{t} \in \mathbb {R}^{T}$:

$$\begin{array}{@{}rcl@{}} r_{i} = \sigma \left(\boldsymbol{W}_{rt}^{\top}\boldsymbol{h}_{t} + \boldsymbol{W}_{ri}^{\top}\boldsymbol{h}_{i} + b_{rt}\right), \end{array} $$

$$\begin{array}{@{}rcl@{}} e_{t,i} = \boldsymbol{W}_{et}^{\top}((1-r_{i}) \odot \boldsymbol{h}_{t} + r_{i} \odot \boldsymbol{h}_{i})+b_{et}, \end{array} $$

$$\begin{array}{@{}rcl@{}} \boldsymbol{\beta}_{t} = \text{Softmax}([e_{t,1}, \cdots, e_{t,i},\cdots, e_{t,T}]), \end{array} $$

where $\boldsymbol {W}_{rt} \in \mathbb {R}^{2q}, \boldsymbol {W}_{ri} \in \mathbb {R}^{2q}, b_{rt} \in \mathbb {R}, \boldsymbol {W}_{et} \in \mathbb {R}^{2q}$, and $b_{et} \in \mathbb {R}$ are the learnable parameters. Finally, a temporal context vector $\boldsymbol {c}_{t} \in \mathbb {R}^{2q}$ can be derived as the output of the time-wise attention:

8 $$\begin{array}{@{}rcl@{}} \boldsymbol{c}_{t}=\sum\limits_{i=1}^{T}\beta_{t,i} \odot \boldsymbol{h}_{t}. \end{array} $$

### Unified neural classifier

With the help of our hybrid attention mechanism, we can obtain an attentional representation $\boldsymbol {\hat {h}} \in \mathbb {R}^{r}$ by fusing the context vector ***c***_*t*_ and the current hidden state ***h***_*t*_, defined as:

$$\begin{array}{@{}rcl@{}} \boldsymbol{\hat{h}}_{t} = f(\boldsymbol{W}_{h}[\boldsymbol{c}_{t} \oplus \boldsymbol{h}_{t}] + \boldsymbol{b}_{h}), \end{array} $$

where $\boldsymbol {W}_{h} \in \mathbb {R}^{r \times 4q}$ and $\boldsymbol {b}_{h} \in \mathbb {R}^{r}$ denote the learnable parameters. The attentional representation is then fed through the softmax layer to classify sleep stages, as follows:

9 $$\begin{array}{@{}rcl@{}}  \boldsymbol{\hat{y}}_{t} = \text{Softmax}(\boldsymbol{W}_{s}\boldsymbol{\hat{h}}_{t}+\boldsymbol{b}_{s}). \end{array} $$

where $\boldsymbol {W}_{s} \in \mathbb {R}^{|\mathcal {C}| \times r}$ and $\boldsymbol {b}_{s} \in \mathbb {R}^{|\mathcal {C}|}$ are the learnable parameters. To train HybridAtt in an end-to-end manner, we employ cross-entropy to measure the classification loss between the $\boldsymbol {\hat {y}}_{t}$ obtained by Eq. 9 and the ground truth ***y***_*t*_. The cost function of our unified HybridAtt model *J*_HybridAtt_ is defined as:

$$\begin{array}{@{}rcl@{}}\small \begin{aligned} & J_{\textsf{\scriptsize HybridAtt}}\left(\boldsymbol{X}_{1}^{(1)},\cdots,\boldsymbol{X}_{T^{(1)}}^{(1)},\cdots,\boldsymbol{X}_{1}^{(M)}, \cdots,\boldsymbol{X}_{T^{(M)}}^{(M)}\right) \\ = & \ -\frac{1}{M} \sum^{M}_{i=1}\frac{1}{T^{(i)}}\sum\limits_{t=1}^{T^{(i)}}\left[ \boldsymbol{y}_{t}^{\top}\log{\boldsymbol{\hat{y}}_{t}}+(\boldsymbol{1}-\boldsymbol{y}_{t})^{\top} \log{(\boldsymbol{1}-\boldsymbol{\hat{y}}_{t})}\right]. \end{aligned} \end{array} $$

## Results and discussion

In this section, we evaluate HybridAtt on a benchmark PSG dataset in two feature domains, referred to as the time and frequency domains. We first introduce the dataset, then describe the baselines and some experiment details. We finally present and discuss the quantitative results in terms of different evaluation metrics.

### Dataset description

We conduct experiments for multivariate PSG sleep stage classification based on the UCD dataset collected from St. Vincent’s University Hospital and University College Dublin [32]. This dataset contains 14-channel overnight PSG data, consisting of 128Hz EEG, 64Hz EMG, and other types of biosignals. We generate 287,840 input vectors from all 25 subjects, and each 30-s fragment is labeled as in one of the five sleep stages. In more detail, a 30-s long timestamp contains 53,760 data points in the time domain, and 27,300 data points in the frequency domain using short-time Fourier transform (STFT). Note that we merge the original S3 and S4 stages as a new S3 stage, and only retain the time slots belonging to the five sleep stages in our experiments.

### Baselines

We compare HybridAtt with the following ten existing biosignal feature learning baselines:

*SVM.* SVM is a classic machine learning method. Here we use one-vs-all SVM for the five-class classification task. To avoid the curse of dimensionality, we utilize principal component analysis (PCA) to select top-*r* related components from all the PSG channels as features before training SVM, namely PSVM.

*Deep neural networks (DNN).* DNN is a basic multi-layer neural network. We train a 3-layer DNN with softmax by concatenating all the PSG channels as input.

*RNN.* RNN is designed for time series. Similar to DNN, we concatenate data and train the same BGRU structure as HybridAtt used with a softmax layer.

*RNNAtt.* RNNAtt is a RNN variant with attention mechanism. We add two existing attention strategies, called location-based and concatenation-based attention [33], after the BGRU structure, referred to as RNNAtt _*l*_ and RNNAtt _*c*_, respectively.

*CNN.* CNN is a commonly used deep learning model for biosignals. We integrate the PSG data as a matrix, and train the same CNN structure in our multi-view convolutional representation module.

*CRNN.* CRNN is a CNN variant combined with RNN. Here we directly integrate the aforementioned CNN and BGRU to train a unified model.

*CRNNAtt.* CRNNAtt utilizes attention mechanism after the CRNN structure. Similarly, we perform the same process as RNNAtt, namely CRNNAtt _*l*_ and CRNNAtt _*c*_, respectively.

*ChannelAtt.* ChannelAtt [34] is proposed to soft-select critical channels from multivariate biosignals using a global attention mechanism. Different from the original model using fully-connected layer for feature extraction, we use the proposed CNN structure as the feature encoder to train the model.

### Our approaches

To fairly evaluate our proposed attention strategy, we show the performance of the following two approaches in the experiments.

*HybridAtt*_*l*_. HybridAtt _*l*_ is a reduced model using the location-based attention mechanism in HybridAtt for sleep stage classification.

*HybridAtt*_*f*_. HybridAtt _*f*_ uses the proposed attention strategy to calculate score vectors in the channel-wise and time-wise layers.

### Evaluation criteria

To quantify the performance, five evaluation measurements are used to validate HybridAtt for PSG-based sleep stage classification. Both accuracy and F1-score are adopted for evaluation. Here we employ Macro and Micro metric to measure F1-score, namely Macro-F1 and Micro-F1, respectively. The Macro-based area-under-the-curve (AUC) of precision-recall (PR) and receiver operator characteristic (ROC) are also utilized to evaluate each approach, namely AUC-PR and AUC-ROC, respectively. Moreover, to evaluate our model as a general cross-subject classifier, we perform 5-fold subject-independent cross validation and report the average test performance with standard deviation (*μ*±*σ*) for each method. The ratio of training, validation and test sets is 0.7:0.1:0.2.

### Implementation details

We implement all the approaches with Pytorch. The training process is done locally using NVIDIA Titan Xp GPU. Adadelta [35] is adopted for the training process to optimize the cost function in terms of the learnable parameters. We also use weight decay with 0.001 L2 penalty coefficient, 0.95 momentum, and 0.5 dropout rate for all the approaches. The structure configuration of our multi-view convolutional representation module is listed in Table 1, and we set *p*=128,*q*=128, and *r*=128 for our models and baselines.

**Table 1:** Configurations of the multi-view convolutional representation module in HybridAtt

Type	Kernel size	Stride	Padding
$Conv1d_{1\_1}$	8×8	2	3
$Conv1d_{1\_2}$	16×8	2	7
$Conv1d_{1\_3}$	32×8	2	3
$Conv1d_{1\_4}$	64×8	2	7
*MaxPool*1*d*_1_	6	4	1
$Conv1d_{2\_1}$	3×16	1	1
$Conv1d_{2\_2}$	5×16	1	2
*MaxPool*1*d*_2_	3	2	1
$Conv1d_{3\_1}$	3×16	1	1
$Conv1d_{3\_2}$	5×16	1	2
*MaxPool*1*d*_3_	3	2	1
$Conv2d_{1\_1}$	1×8×8	1,2	0,3
$Conv2d_{1\_2}$	1×16×8	1,2	0,7
$Conv2d_{1\_3}$	1×32×8	1,2	0,3
$Conv2d_{1\_4}$	1×64×8	1,2	0,7
*AvgPool*2*d*_1_	1×6	1,4	0,1
$Conv2d_{2\_1}$	3×3×16	1,1	1,1
$Conv2d_{2\_2}$	5×5×16	1,1	2,2
*AvgPool*2*d*_2_	1×3	1,2	0,1
$Conv2d_{3\_1}$	3×3×16	1,1	1,1
$Conv2d_{3\_2}$	5×5×16	1,1	2,2
*AvgPool*2*d*_3_	14×3	14,2	0,1

### Experimental results

We investigate the effectiveness of our proposed HybridAtt model, compared to the aforementioned baseline methods in the task of sleep stage classification. Tables 2 and 3 report the comparison results tested in the frequency and time domains, respectively. We highlight the best evaluation scores in boldface. We observe that HybridAtt achieves the best performance compared with the corresponding baselines in both feature domains on the UCD dataset.

**Table 2:** Classification performance comparisons on the UCD dataset in the frequency domain

	UCD Dataset (frequency Domain)				
Method	AUC-ROC	AUC-PR	Macro-F1	Micro-F1	Accuracy
PSVM	0.8177 ±0.0142	0.5767 ±0.0172	0.5204 ±0.0275	0.5854 ±0.0733	0.6193 ±0.1053
DNN	0.7213 ±0.1435	0.5224 ±0.1048	0.3542 ±0.2171	0.4331 ±0.2269	0.5262 ±0.1613
RNN	0.6228 ±0.0465	0.3350 ±0.0394	0.2663 ±0.0241	0.3970 ±0.0428	0.5091 ±0.0391
RNNAtt _*l*_	0.6172 ±0.0386	0.3305 ±0.0386	0.2457 ±0.0307	0.3734 ±0.0566	0.5002 ±0.0476
RNNAtt _*c*_	0.6234 ±0.0451	0.3335 ±0.0345	0.2554 ±0.0258	0.3712 ±0.0325	0.5010 ±0.0367
CNN	0.8732 ±0.0129	0.6725 ±0.0120	0.5925 ±0.0604	0.6492 ±0.0841	0.6590 ±0.0979
CRNN	0.8660 ±0.0074	0.6454 ±0.0135	0.5693 ±0.0060	0.6395 ±0.0370	0.6634 ±0.0412
CRNNAtt _*l*_	0.8570 ±0.0183	0.6281 ±0.0359	0.5810 ±0.0371	0.6486 ±0.0641	0.6683 ±0.0657
CRNNAtt _*c*_	0.8671 ±0.0274	0.6418 ±0.0401	0.5849 ±0.0577	0.6528 ±0.0547	0.6791 ±0.0546
ChannelAtt	0.8705 ±0.0483	0.6818 ±0.0580	0.6517 ±0.0334	0.7070 ±0.0605	0.7152 ±0.0574
HybridAtt _*l*_	0.8719 ±0.0214	0.6669 ±0.0297	0.6342 ±0.0316	0.6962 ±0.0645	0.7070 ±0.0707
HybridAtt _*f*_	**0.8854** ±**0.0137**	**0.6886** ±**0.0256**	**0.6639** ±**0.0301**	**0.7231** ±**0.0489**	**0.7328** ±**0.0546**

**Table 3:** Classification performance comparisons on the UCD dataset in the time domain

	UCD Dataset (Time Domain)				
Method	AUC-ROC	AUC-PR	Macro-F1	Micro-F1	Accuracy
PSVM	0.4945 ±0.0068	0.2249 ±0.0042	0.1352 ±0.0388	0.2584 ±0.0464	0.3877 ±0.1107
DNN	0.5024 ±0.0025	0.5128 ±0.0144	0.1129 ±0.0282	0.2372 ±0.1023	0.3962 ±0.1193
RNN	0.6236 ±0.0424	0.3352 ±0.0358	0.2464 ±0.0360	0.3417 ±0.0908	0.4487 ±0.1174
RNNAtt _*l*_	0.6256 ±0.0297	0.3254 ±0.0279	0.2557 ±0.0328	0.3483 ±0.0938	0.4521 ±0.1100
RNNAtt _*c*_	0.6279 ±0.0549	0.3434 ±0.0376	0.2465 ±0.0314	0.3328 ±0.0870	0.4501 ±0.1087
CNN	0.8421 ±0.0186	0.5844 ±0.0300	0.5775 ±0.0336	0.6493 ±0.0326	0.6595 ±0.0347
CRNN	0.8453 ±0.0229	0.5945 ±0.0297	0.5761 ±0.0345	0.6483 ±0.0383	0.6592 ±0.0456
CRNNAtt _*l*_	0.8461 ±0.0115	0.6097 ±0.0206	0.5954 ±0.0362	0.6585 ±0.0437	0.6659 ±0.0473
CRNNAtt _*c*_	0.8505 ±0.0140	0.6120 ±0.0273	0.6004 ±0.0319	0.6622 ±0.0509	0.6632 ±0.0541
ChannelAtt	0.8720 ±0.0203	0.6834 ±0.0278	0.6107 ±0.0324	0.6907 ±0.0545	0.7169 ±0.0624
HybridAtt _*l*_	0.8885 ±0.0142	0.7009 ±0.0223	0.6689 ±0.0314	0.7264 ±0.0491	0.7317 ±0.0512
HybridAtt _*f*_	0.8966 ±**0.0214**	**0.7082** ±**0.0283**	**0.6818** ±**0.0304**	**0.7368** ±**0.0591**	**0.7424** ±**0.0594**

Given the results of the baselines, the performance of the traditional classification method PSVM is better than DNN and the RNN-based models in the frequency domain, but worse in the time domain. It means that the raw frequency features of PSG data would carry distinctive information which help SVM learn relatively clear hyper-lane to separate sleep stages. The results of DNN between two feature domains also make the same observation, demonstrating the capability of the PSG spectral features. The limited improvement of attention-based RNN models, compared with RNN in both domains, show that the features learned by RNN does not provide enough information for attention mechanisms to make correct classification. This also indicates that simply concatenating PSG data is unsuitable for fully-connected networks to learn informative features, since it would ignore multivariate prior information. We can see that CNN-based models get better performance than the other baselines, benefiting from the proposed structure in the multi-view convolutional representation module. Compared with CRNN, the attention-based CRNN models perform better, because attention mechanism is able to fuse features based on more useful information carried by sequential representations. To fuse features from multi-channel representations using attention mechanism, ChannelAtt works well in classifying sleep stages and achieve better results than CRNNAtt. It illustrates that the hidden connections among PSG channels, captured by ChannelAtt, are more helpful for sleep stage classification. Furthermore, by capturing dual correlations among channels and timestamps, our proposed HybridAtt model consistently gains the best evaluation scores in both the time and frequency feature domains.

From the results of our models, HybridAtt _*f*_ outperforms the baselines in terms of all five evaluation measurements. For example, HybridAtt _*f*_ obtains the best accuracy of 0.7424 in the time domain, compared with 0.7317 and 0.7169 achieved by our reduced model HybridAtt _*l*_ and the baseline model ChannelAtt, respectively. Compared the results between two domains, on one hand, we observe that the results of HybridAtt _*l*_ performs on par with those of ChannelAtt in the frequency domain. It means that adopting traditional location-based attention in the channel-wise layer cannot capture enough information from multi-view representations, and hence fail to help time-wise attention extract high-level features. On the other hand, HybridAtt _*f*_, utilizing the proposed attention strategy fuse multi-view features, achieves a robust performance under different raw feature spaces. Moreover, the results of HybridAtt in the time domain performs better than those in the frequency domain. We conjecture that CNN has a similar convolution procedure as STFT, but CNN adopts learnable kernels during convolution while STFT employs fixed Fourier functions. Taking the advantage of end-to-end learning, our hybrid attention mechanism can help learn more representative convolutional kernels in CNN than the handcrafted window functions in STFT.

**Fig. 3:**
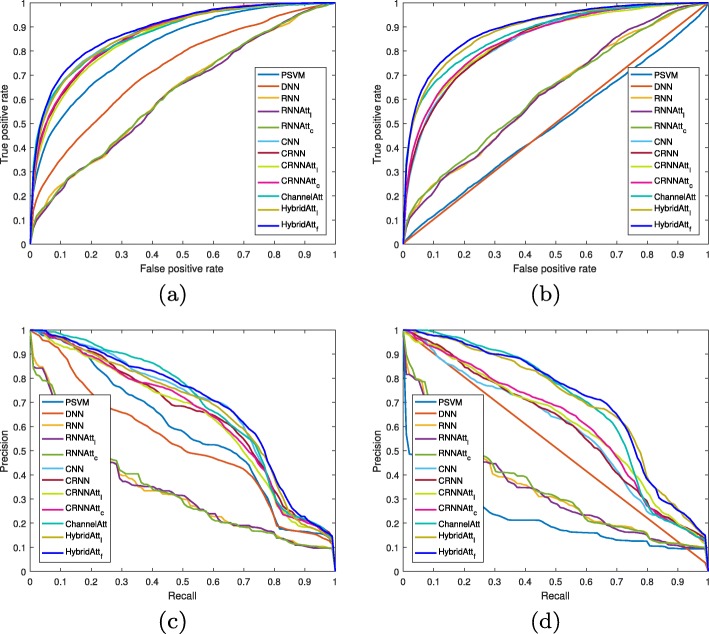
ROC and PR curves of the proposed method and the baselines in different feature domains on the UCD dataset. **a** and **b** plots the ROC curves in the frequency and time domains, respectively. **c** and **d** plots the PR curves in the frequency and time domains, respectively

Figure 3 illustrates the ROC and PR curves of all the test folds on the UCD dataset, respectively. We observe that the proposed HybridAtt _*f*_ method consistently gains the best AUC in terms of the PR and ROC in different domains, demonstrating an effective cross-subject method in the task of sleep stage classification. Based on the overall performance comparisons, we conclude that attention mechanism is key to identify sleep patterns for sleep stage classification. Adopting single-dimension attention in different aspects, such as CRNNAtt and ChannelAtt, may lose useful information dealing with multivariate PSG records. Multi-view representation is also essential for attention mechanism inferring important information. By constructing hybrid attention networks based on multi-view convolutional representation, the HybridAtt achieves better results in both feature domains, in comparison with different feature learning methods, demonstrating the effectiveness of HybridAtt in PSG-based sleep stage classification.

## Conclusions

In this paper, we present a unified hybrid self-attention deep learning framework, namely HybridAtt, to classify sleep stages from multivariate PSG records. HybridAtt is designed to capture dual correlations among channels and timestamps based on multi-view convolutional feature representations. Experiments on a benchmark PSG dataset show that HybridAtt is able to efficiently fuse multivariate information from PSG data and hence consistently beats the baselines in both the time and the frequency feature domains. In future work, we will extend HybridAtt to other biomedical applications with similar data structure, and propose advanced attention mechanism that can jointly learn two-dimensional contribution scores in one step, instead of adopting the multi-step attention strategy.

## Data Availability

The UCD dataset used in our experiments can be downloaded in https://physionet.org/physiobank/database/ucddb/. The data is available for public and free to use.

## Abbreviations

AUC: Area under the curve
BGRU: Bidirectional gated recurrent units
CNN: Convolutional neural networks
DBN: Deep belief networks
DNN: Deep neural networks
ECG: Electrocardiogram
EEG: Electroencephalogram
EMG: Electromyogram
EOG: Electrooculogram
NN: Neural networks
NREM: Non-rapid eye movement
PCA: Principal component analysis
PR: Precision recall
PSG: Polysomnography
R&K: Rechtschaffen and Kales
RNN: Recurrent neural networks
ROC: Receiver operator characteristic
STFT: Short-time fourier transform
SVM: Support vector machine

## References

[1] Luyster FS, Strollo PJ, Zee PC, Walsh JK (2012). Sleep: a health imperative. Sleep.

[2] Aboalayon KAI, Faezipour M, Almuhammadi WS, Moslehpour S (2016). Sleep stage classification using eeg signal analysis: a comprehensive survey and new investigation. Entropy.

[3] Boostani R, Karimzadeh F, Nami M (2017). A comparative review on sleep stage classification methods in patients and healthy individuals. Comput Methods Programs Biomed.

[4] Şen B, Peker M, Çavuşoğlu A, Çelebi FV (2014). A comparative study on classification of sleep stage based on eeg signals using feature selection and classification algorithms. J Med Syst.

[5] Wolpert EA (1969). A manual of standardized terminology, techniques and scoring system for sleep stages of human subjects. Arch Gen Psychiatr.

[6] Khalighi S, Sousa T, Oliveira D, Pires G, Nunes U. Efficient feature selection for sleep staging based on maximal overlap discrete wavelet transform and svm. In: Engineering in Medicine and Biology Society, EMBC, 2011 Annual International Conference of the IEEE. IEEE: 2011. p. 3306–9. 10.1109/IEMBS.2011.6090897 22255046

[7] Tsai P-Y, Hu W, Kuo TB, Shyu L-Y. A portable device for real time drowsiness detection using novel active dry electrode system. In: Engineering in Medicine and Biology Society, 2009. EMBC 2009. Annual International Conference of the IEEE. IEEE: 2009. p. 3775–8. 10.1109/IEMBS.2009.5334491 19964814

[8] Charbonnier S, Zoubek L, Lesecq S, Chapotot F (2011). Self-evaluated automatic classifier as a decision-support tool for sleep/wake staging. Comput Biol Med.

[9] Li Y, Yingle F, Gu L, Qinye T. Sleep stage classification based on eeg hilbert-huang transform. In: Industrial Electronics and Applications, 2009. ICIEA 2009. 4th IEEE Conference On. IEEE: 2009. p. 3676–81. 10.1109/iciea.2009.5138842.

[10] Shi J, Liu X, Li Y, Zhang Q, Li Y, Ying S (2015). Multi-channel eeg-based sleep stage classification with joint collaborative representation and multiple kernel learning. J Neurosci Methods.

[11] Phan H, Do Q, Do T-L, Vu D-L. Metric learning for automatic sleep stage classification. In: Engineering in Medicine and Biology Society (EMBC), 2013 35th Annual International Conference of the IEEE. IEEE: 2013. p. 5025–8. 10.1109/embc.2013.6610677. 24110864

[12] Huang C-S, Lin C-L, Ko L-W, Liu S-Y, Sua T-P, Lin C-T. A hierarchical classification system for sleep stage scoring via forehead eeg signals. In: Computational Intelligence, Cognitive Algorithms, Mind, and Brain (CCMB), 2013 IEEE Symposium On. IEEE: 2013. p. 1–5.

[13] Gudmundsson S, Runarsson TP, Sigurdsson S. Automatic sleep staging using support vector machines with posterior probability estimates. In: Computational Intelligence for Modelling, Control and Automation, 2005 and International Conference on Intelligent Agents, Web Technologies and Internet Commerce, International Conference On, vol. 2. IEEE: 2005. p. 366–72. 10.1109/cimca.2005.1631496.

[14] Özşen S (2013). Classification of sleep stages using class-dependent sequential feature selection and artificial neural network. Neural Comput Applic.

[15] Tagluk ME, Sezgin N, Akin M (2010). Estimation of sleep stages by an artificial neural network employing eeg, emg and eog. J Med Syst.

[16] Najdi Shirin, Gharbali Ali Abdollahi, Fonseca José Manuel (2017). Feature Transformation Based on Stacked Sparse Autoencoders for Sleep Stage Classification. IFIP Advances in Information and Communication Technology.

[17] Längkvist M, Karlsson L, Loutfi A (2012). Sleep stage classification using unsupervised feature learning. Adv Artif Neural Syst.

[18] Zhang J, Wu Y, Bai J, Chen F (2016). Automatic sleep stage classification based on sparse deep belief net and combination of multiple classifiers. Trans Inst Meas Control.

[19] Supratak A, Dong H, Wu C, Guo Y (2017). Deepsleepnet: a model for automatic sleep stage scoring based on raw single-channel eeg. IEEE Trans Neural Syst Rehabil Eng.

[20] Tsinalis O, Matthews PM, Guo Y, Zafeiriou S. Automatic sleep stage scoring with single-channel eeg using convolutional neural networks. 2016. arXiv preprint arXiv:1610.01683.

[21] Chambon S, Galtier MN, Arnal PJ, Wainrib G, Gramfort A (2018). A deep learning architecture for temporal sleep stage classification using multivariate and multimodal time series. IEEE Trans Neural Syst Rehabil Eng.

[22] Giri EP, Fanany MI, Arymurthy AM. Combining generative and discriminative neural networks for sleep stages classification. 2016. arXiv preprint arXiv:1610.01741.

[23] Zhao M, Yue S, Katabi D, Jaakkola TS, Bianchi MT. Learning sleep stages from radio signals: a conditional adversarial architecture. In: International Conference on Machine Learning. ACM: 2017. p. 4100–9.

[24] Guilleminault C, Tilkian A, Dement WC (1976). The sleep apnea syndromes. Annu Rev Med.

[25] Thorpy Michael J. (1990). Classification of Sleep Disorders. Journal of Clinical Neurophysiology.

[26] Zhao J, Xie X, Xu X, Sun S (2017). Multi-view learning overview: Recent progress and new challenges. Inform Fusion.

[27] Yuan Y, Xun G, Jia K, Zhang A. A multi-view deep learning method for epileptic seizure detection using short-time fourier transform. In: Proceedings of the 8th ACM International Conference on Bioinformatics, Computational Biology, and Health Informatics. ACM: 2017. p. 213–22. 10.1145/3107411.3107419.

[28] Yuan Y, Xun G, Jia K, Zhang A (2018). A multi-context learning approach for eeg epileptic seizure detection. BMC Syst Biol.

[29] Yuan Y, Jia K, Ma F, Xun G, Wang Y, Su L, Zhang A. Multivariate sleep stage classification using hybrid self-attentive deep learning networks. In: 2018 IEEE International Conference on Bioinformatics and Biomedicine (BIBM). IEEE: 2018. p. 963–8. 10.1109/bibm.2018.8621146.

[30] Yuan Y, Xun G, Suo Q, Jia K, Zhang A (2018). Wave2vec: Deep representation learning for clinical temporal data. Neurocomputing.

[31] Schuster M, Paliwal KK (1997). Bidirectional recurrent neural networks. IEEE Trans Signal Process.

[32] Goldberger AL, Amaral LA, Glass L, Hausdorff JM, Ivanov PC, Mark RG, Mietus JE, Moody GB, Peng C-K, Stanley HE (2000). Physiobank, physiotoolkit, and physionet. Circulation.

[33] Ma F, Chitta R, Zhou J, You Q, Sun T, Gao J. Dipole: Diagnosis prediction in healthcare via attention-based bidirectional recurrent neural networks. In: Proceedings of the 23rd ACM SIGKDD International Conference on Knowledge Discovery and Data Mining. ACM: 2017. p. 1903–11. 10.1145/3097983.3098088.

[34] Yuan Y, Xun G, Ma F, Suo Q, Xue H, Jia K, Zhang A. A novel channel-aware attention framework for multi-channel eeg seizure detection via multi-view deep learning. In: Biomedical & Health Informatics (BHI), 2018 IEEE EMBS International Conference On. IEEE: 2018. p. 206–9. 10.1109/bhi.2018.8333405.

[35] Zeiler MD. Adadelta: an adaptive learning rate method. 2012. arXiv preprint arXiv:1212.5701.

